# A video review of multiple concussion signs in National Rugby League match play

**DOI:** 10.1186/s40798-017-0117-9

**Published:** 2018-01-12

**Authors:** Andrew J. Gardner, David R. Howell, Grant L. Iverson

**Affiliations:** 1 Centre for Stroke and Brain Injury, School of Medicine and Public Health, University of Newcastle, Newcastle, NSW Australia; 2 Hunter New England Local Health District Sports Concussion Program, John Hunter Hospital, Newcastle, NSW Australia; 3 Department of Orthopedics, Sports Medicine Center, Children’s Hospital Colorado, University of Colorado School of Medicine, Aurora, CO USA; 4 Department of Physical Medicine and Rehabilitation, Harvard Medical School, Boston, MA USA; 5 Spaulding Rehabilitation Hospital, Boston, MA USA; 6 MassGeneral Hospital for Children™ Sport Concussion Program, Boston, MA USA; 7 Home Base, A Red Sox Foundation and Massachusetts General Hospital Program, Boston, MA Australia

**Keywords:** Concussion, Video analysis, Injury management, Return to play

## Abstract

**Background:**

Video review has been introduced in many professional sports worldwide to help recognize concussions. However, to date, there has been very little research on the accuracy of using video analysis to identify signs of concussion and the various combinations of observed signs.

**Methods:**

The objective of the study is to determine the accuracy of combinations of clinical signs of concussion identified using video analysis to identify concussions in the National Rugby League (NRL). Incidences of players using of the concussion interchange rule (CIR) (*n* = 156), including those where athletes were diagnosed with a concussion (*n* = 60), were used to calculate sensitivity and specificity of various combinations of concussion signs (unresponsiveness, slow to get up, clutching/shaking head, gait ataxia, vacant stare, and apparent seizure) and their independent association with an eventual diagnosis of concussion.

**Results:**

Using video analysis, players who were diagnosed with a concussion showed a significantly greater total number of signs at the time of injury (mean = 3.4, SD = 1.3) than those who were removed from play but not diagnosed with a concussion (mean = 3.0, SD = 0.9 signs; *p* = .046). Players who did not return to play during the same game demonstrated a significantly greater number of total signs than those who did return to play in the same game following CIR activation (mean = 3.4, SD = 1.2 versus mean = 2.9, SD = 0.9; *p* = 0.002). The most common *combination* of signs that was observed was clutching/shaking the head and slowness in getting up (17.3%). The sensitivity of the total number of signs observed decreased as the number of signs increased (range = 0.13–0.62), while the specificity increased as more signs were observed (range = 0.29–0.90). Most of the combinations of different observed signs at the time of potential injury were highly specific (> 0.80), but not sensitive to an eventual diagnosis of concussion. When considering all potential predictor variables in a logistic regression model, anticipating the oncoming collision (OR = 3.92, 95% CI = 1.28–12.03), fewer number of defenders involved in the tackle (OR = 0.58, 95% CI = 0.36–0.92), and the presence of a blank or vacant stare (OR = 2.97, 95% CI = 1.26–7.01) were each significantly associated with concussion diagnoses.

**Conclusions:**

The use of video review in the NRL is challenging, but being aware of the combinations of possible concussion signs and the likelihood that various presentations result in a concussion diagnosis can provide a useful addition to sideline concussion identification and removal from play decisions.

## Key points

- Video replay analysis is useful for sports medicine professionals, but it also can be difficult to interpret and presents challenges in identifying those who have sustained a concussion.
- Signs and combinations of signs have limited accuracy as indicators of concussion.
- Being aware of the combinations of possible concussion signs and the likelihood that various presentations result in a concussion diagnosis can provide a useful addition to sideline concussion identification.
- The most common *combination* of signs that was observed was clutching/shaking the head and slowness in getting up (17.3%).

## Background

Over the past few years, sideline video review and analysis has become common practice. Its use ranges from professional leagues worldwide to some National Collegiate Athletic Association (NCAA) conferences, with the goal of improving the recognition of a possible concussion [13]. Several studies have reviewed the usefulness and limitations of sideline and post-game video review [2, 5–7, 12, 13]. Our group [2–4] and others [1–3, 13, 14] have conducted several video reviews in professional leagues and reported that video injury surveillance can be difficult to interpret but may provide a useful adjunct to the recognition of concussion [6]. For example, although the presence of a sign after a concussion is sensitive to identifying the injury when reviewing known or suspected injuries, when reviewing an entire season of match play, some concussion signs occur commonly but do not typically reflect the presence of a concussion [4].

Because athletes may demonstrate several signs of a concussion simultaneously following a head impact, one way to maximize the application of sideline video review systems is to examine the risk for concussion associated with the presence of various *combinations* of concussion signs observed with video replay. In addition, reviewing these combinations of signs in the context of match play characteristics (such as anticipating the oncoming impact, tackler versus ball carrier, tackle height, and the number of defenders involved in the tackle) may also assist with maximizing the usefulness of sideline video review systems. For example, in professional hockey, a risk prediction model using a combination of visual signs and mechanism of injury improved the ability to identify players who may require further evaluation for possible concussion [1]. The current study builds on the work that examined the rate of six concussion signs during the 2014 National Rugby League (NRL) season [4–7], by evaluating various combinations of signs for predicting cases of medically diagnosed concussion. The purposes of this study were to determine (i) the combination of observable signs occurring in the NRL using video review and (ii) the combination of observable signs and match play characteristics that were associated with a concussion diagnosis when the concussion interchange rule (CIR) was activated.

## Methods

### Participants and procedures

The professional rugby league governing body in Australia introduced a new CIR for the 2014 NRL season. The CIR allows a player suspected of having sustained a concussion to be removed from play and assessed without an interchange being tallied against the player’s team. In this study, we conducted a video analysis of footage when the CIR was activated during the 2014 NRL season. The digital records of all matches involving the use of the CIR were reviewed using the Quicktime Multimedia Player V.7.7.5. We reviewed the entire play that led to each CIR activation throughout the season. The six signs that were identified consisted of clutching or shaking the head, unresponsiveness or loss of consciousness, slowness to get up, gait ataxia, vacant stare, and apparent seizure.

The videos of each incident where players were removed under the CIR (i.e., suspected of having sustained a concussion during match play) were reviewed by a single investigator. The CIR includes a 15-min assessment window for a club medical officer to complete the Sports Concussion Assessment Tool 3rd edition (SCAT-3) and any other assessment deemed necessary to determine if a concussion occurred. Following this assessment, if the player is cleared to return to play within the 15-min assessment period, the team is not penalized an interchange (i.e., the team receives a “free interchange”). If the player is cleared to return to play outside of this 15-min assessment window, the team is charged with an interchange. In the event that an athlete is not cleared to return to play, the interchange is not tallied against the injured player’s team.

The club physician made the final diagnosis of concussion based on conventional clinical examination techniques. For every CIR activation, cases were reviewed for a medically diagnosed concussion using the Observational Review and Analysis of Concussion (ORAC) Form to record the game circumstances and detail the suspected injury and the presence or absence of the six concussive signs [8]. The ORAC Form was created to provide a simple but standardized framework for coding and analyzing video footage of the game situations and consequences of concussion events in rugby league [8]. In summary, the six signs included the following: (i) clutch or shake head: the player holds his head or face in the palm of his hand or hands, or the player rubs or shakes his head in a manner that appears to demonstrate that he is experiencing discomfort; (ii) slow to return to feet/play: player took longer than usual to return to his feet (e.g., remained on the ground, got to his knees or to his haunches, and waited momentarily before standing); (iii) gait ataxia (wobbly legs): unable to stand steadily unaided or walk normally. Upon standing and walking, the player has unsteadiness, wobbly legs, balance problems, stumbles or falls over, or cannot walk straight independently; (iv) blank/vacant stare: the player is not visually focused on the doctor/trainer when being spoken to or assessed and asked to attend, and/or the player appears to be looking off into the distance; (v) evidence of unresponsiveness: the player’s body goes limp, the player *does not* protect (i.e., brace) himself when falling. The player remains/lies motionless on the ground for a period of time longer than expected, or the player shows a lack of visible responsiveness to verbal stimuli. The sign was recorded as present even if the player exhibited behavior that met the criteria for only 1 sec; and (vi) post-impact seizure or possible seizure: tonic posturing—stiffening of limbs or convulsions [9]. The inter-rater reliability (IRR) for each of these signs has been previously reported [4, 6]. The reported IRR for two expert raters for each sign was clutch or shake head, slow to get up, and post-impact seizure = 1.00; unresponsiveness = 0.84 (95% CI: 0.70–0.94); gait ataxia = 0.77 (95% CI: 0.63–0.90); and blank stare = 0.65 (95% CI: 0.44–0.84) [4].

For this study, the operational definition of the variable “anticipation of hit” included the following: (i) any player who initiated the impact himself, (ii) any player who could visibly observe an imminent impact, or (iii) any player who physically braced himself for the impact. This study was approved by the University of Newcastle Human Ethics Committee (H-2012-0344).

### Statistical analysis

Categorical variables are presented as percentages and were assessed using Fisher’s exact test, and continuous variables are presented as means (standard deviation) and compared using independent sample *t* tests. Sensitivity, specificity, positive predictive value, and negative predictive values were calculated according to (a) the total number of signs observed (i.e., two or more, three or more, four or more) and (b) each of the different combinations of observed signs documented during the study period. These calculations were done using 2 × 2 contingency tables. In addition to frequency distributions, we evaluated potential predictor variables between the cases that led to a medically diagnosed concussion versus those cases that did not when the CIR was activated. Variables that appeared different between the two groups with a statistical probability of *p* < 0.20 were identified as potential predictor variables and considered for inclusion in a logistic regression model. Prior to performing logistic regression analyses, we assessed collinearity with condition indices and variance inflation factors. If a condition index was greater than 30, individual collinearity assessments were conducted using variance inflation factors; those > 2.5 [15] were identified as collinear, and only one variable was placed into the model. Statistical significance was determined with a threshold of *p* < 0.05, or for the logistic regression model, an adjusted odds ratio (aOR) with a 95% confidence interval not containing one. Statistical analyses were performed with Statistical Package for the Social Sciences (SPSS version 23, IBM Inc., Armonk, NY, USA).

## Results

During the 2014 season, there were 167 observed incidences where the CIR was used, and we located and reviewed 162 (97%). Of these cases, return to play data was available for 156 (96%) of incidences, which was the final number of cases used in our analyses. Of these events, athletes were subsequently medically diagnosed with a concussion 38% of the time by their club physician (*n* = 60). All 60 cases of medically diagnosed concussion are included in the analysis, along with the 96 cases where the CIR was used but the athlete was not diagnosed with a concussion following the suspected event.

The majority of times the interchange rule was used occurred as a result of a “hit-up” type of play (*n* = 99), while plays involving the backline (*n* = 8), a backline break (*n* = 7), and straightening up the line of an attacking run (*n* = 6) were also plays that resulted in interchange use. A significantly greater number of total signs were observed at the time of suspected injury among those who were subsequently medically diagnosed with a concussion than those who were not, while those who did not return to play during the same game demonstrated a significantly greater number of total signs than those who did return to play in the same game (Table 1). Not all signs were identified in every incident due to variability in the quality of the available video footage. The amount of missing data for each sign was as follows: vacant stare *n* = 18, gait ataxia *n* = 18, unresponsiveness *n* = 2, and clutching or shaking head *n* = 1. The most common *combinations* of signs that were observed were clutching/shaking the head and slowness in getting up (17.3%); clutching/shaking the head, slowness in getting up, and a vacant stare (13.5%); and clutching/shaking the head, slowness in getting up, and gait ataxia (10.9%; Table 2). The sensitivity of the total number of signs observed decreased as the number of signs increased (range = 0.13–0.62), while the specificity increased as more signs were observed (range = 0.29–0.90; Table 3). Most of the combinations of different observed signs at the time of potential injury were highly specific (> 0.80) but not sensitive to an eventual diagnosis of concussion (< 0.20; Table 2).

**Table 1:** The total number of concussion signs observed during the event, comparing those who were/were not diagnosed with a concussion and those who did/did not return to play

		Total number of signs	*p* value	Cohen’s d effect size
Medically diagnosed with concussion?	Yes (*n* = 60)	3.4 (1.3)	.046	0.35
	No (*n* = 96)	3.0 (0.9)		
Returned to play during the game?	Yes (*n* = 80)	2.9 (0.9)	.002	0.50
	No (*n* = 76)	3.4 (1.2)

**Table 2:** Frequency, sensitivity, specificity, positive predictive value, and negative predictive value of the different combinations of signs demonstrated during the study period

Combination of signs observed	*N* (%)	Sensitivity (95% CI)	Specificity (95% CI)	Positive predictive value (95% CI)	Negative predictive value (95% CI)
Clutch/shake head + slow to get up	27 (17.3)	0.20 (0.12–0.28)	0.84 (0.80–0.90)	0.44 (0.27–0.63)	0.63 (0.59–0.67)
Clutch/shake head + slow to get up + vacant stare	21 (13.5)	0.13 (0.07–0.21)	0.87 (0.82–0.91)	0.38 (0.20–0.60)	0.62 (0.59–0.65)
Clutch/shake head + slow to get up + gait ataxia	17 (10.9)	0.10 (0.04–0.17)	0.89 (0.85–0.93)	0.35 (0.31–0.46)	0.61 (0.59–0.64)
Unresponsiveness + clutch/shake head + slow to get up + vacant stare + gait ataxia	15 (9.6)	0.10 (0.05–0.17)	0.91 (0.87–0.95)	0.40 (0.18–0.66)	0.62 (0.59–0.65)
Unresponsiveness + slow to get up + vacant stare + gait ataxia	14 (9.0)	0.07 (0.02–0.13)	0.90 (0.87–0.94)	0.29 (0.10–0.57)	0.61 (0.59–0.63)
Clutch/shake head + slow to get up + vacant stare + gait ataxia	14 (9.0)	0.07 (0.02–0.13)	0.90 (0.87–0.94)	0.29 (0.10–0.57)	0.61 (0.59–0.63)
Slow to get up + vacant stare + gait ataxia	12 (7.7)	0.07 (0.02–0.13)	0.92 (0.89–0.96)	0.33 (0.12–0.64)	0.61 (0.59–0.64)
Unresponsiveness + clutch/shake head + slow to get up + vacant stare	9 (5.8)	0.03 (0.01–0.09)	0.93 (0.91–0.96)	0.22 (0.04–0.59)	0.61 (0.59–0.63)
Unresponsiveness + slow to get up + gait ataxia	5 (3.2)	0.02 (0.00–0.06)	0.96 (0.95–0.98)	0.20 (0.01–0.70)	0.61 (0.60–0.63)
Slow to get up + vacant stare	4 (2.6)	0.02 (0.00–0.05)	0.97 (0.96–0.99)	0.25 (0.01–0.78)	0.61 (0.61–0.63)
Clutch/shake head + gait ataxia	3 (1.9)	0.05 (0.02–0.05)	1.00 (0.98–1.00)	1.00 (0.31–1.00)	0.63 (0.61–0.63)
Slow to get up + gait ataxia	3 (1.9)	0.02 (0.00–0.04)	0.98 (0.97–1.00)	0.33 (0.02–0.87)	0.61 (0.61–0.63)
Unresponsiveness + seizure + clutch/shake head + slow to get up + vacant stare + gait ataxia	3 (1.9)	0.02 (0.00–0.04)	0.98 (0.97–1.00)	0.33 (0.02–0.87)	0.61 (0.61–0.63)
Unresponsiveness + gait ataxia	2 (1.3)	0.02 (0.00–0.03)	0.99 (0.98–1.00)	0.50 (0.03–0.97)	0.62 (0.61–0.62)
Unresponsiveness + slow to get up + vacant stare	1 (0.6)	0.00 (0.00–0.00)	0.99 (0.99–1.00)	0.00 (0.00–0.95)	0.61 (0.61–0.62)
Unresponsiveness + clutch/shake head + slow to get up + gait ataxia	1 (0.6)	0.00 (0.00–0.00)	0.99 (0.99–1.00)	0.00 (0.00–0.95)	0.61 (0.61–0.62)

Note: There were five incidences where “slow to get up” was the only observed sign

**Table 3:** Sensitivity, specificity, positive predictive value, and negative predictive value of the total number of observed signs (two or more, three or more, four or more) that led to the diagnosis of concussion

	2 or more total observed signs	3 or more total observed signs	4 or more total observed signs
Number of signs present, concussion present	37	18	8
Number of signs present, concussion absent	75	38	10
Number of signs absent, concussion present	23	42	52
Number of signs absent, concussion absent	21	58	86
Sensitivity (95% CI)	0.62 (0.52–0.71)	0.30 (0.21–0.40)	0.13 (0.07–0.20)
Specificity (95% CI)	0.22 (0.16–0.28)	0.60 (0.55–0.67)	0.90 (0.86–0.94)
Positive predictive value (95% CI)	0.33 (0.28–0.38)	0.32 (0.22–0.43)	0.44 (0.23–0.68)
Negative predictive value (95% CI)	0.48 (0.35–0.61)	0.58 (0.52–0.64)	0.62 (0.60–0.65)

Among the predictor variables investigated, those that met criteria for inclusion in the multivariable logistic regression model included the following: positive anticipation of the oncoming hit, the number of defenders involved in the tackle, clutching or shaking the head, unresponsiveness or loss of consciousness, vacant stare, and possible seizure (Table 4). Few observations of a seizure prohibited its inclusion in the logistic regression model. During those events where the CIR was used, anticipating the oncoming hit, the number of defenders involved in the tackle (i.e., fewer), and the presence of a vacant stare were independently associated with eventual diagnosis of concussion (Table 5) after adjusting for the independent effect of all variables included in the model.

**Table 4:** Comparisons of potential predictor variables for those who did and did not receive a diagnosis of concussion following the use of the CIR

Predictor variable	Concussion diagnosis (*n* = 60)	No concussion diagnosis (*n* = 96)	*p* value
	Observed sign: *N* (%) present		
Unresponsiveness or possible loss of consciousness*	24 (40)	26 (27)	.111
Gait ataxia	35 (58)	52 (54)	.484
Vacant stare*	45 (75)	48 (50)	.009
Clutch or shake head*	38 (63)	72 (75)	.105
Slow to get up	60 (100)	93 (97)	.285
Seizure*	3 (5)	0 (0)	.055
	Injury characteristic: *N* (%) present		
Anticipated the oncoming hit*	53 (88)	73 (76)	.063
Low tackle height	11 (18)	18 (19)	.888
	Athlete characteristics: mean (SD)		
Age (years)	26.2 (3.6)	26.6 (3.6)	.487
Injured athlete height (cm)	186.2 (5.1)	186.2 (5.3)	.975
Injured athlete mass (kg)	101.3 (8.5)	100.8 (9.0)	.694
Defenders involved in the tackle (number)*	2.1 (0.9)	2.3 (0.8)	.078
Primary tackler height (cm)	186.9 (5.1)	186.1 (5.9)	.351
Primary tackler mass (kg)	102.5 (9.0)	102.5 (10.9)	.965

*Potential predictor variables considered for inclusion in the logistic regression model (*p* < .20)

**Table 5:** Results for the multivariable logistic regression analysis of the characteristics independently associated with a subsequent diagnosis of concussion

					95% confidence interval for odds ratio	
Predictors	β	Standard error	Wald	Odds ratio^a^	Lower bound	Upper bound
Unresponsiveness or loss of consciousness	− 0.271	0.415	0.426	0.763	0.339	1.719
Vacant stare*	1.090	0.438	6.199	2.974	1.261	7.012
Clutch or shake head	0.450	0.411	1.199	1.569	0.701	3.512
Anticipated the oncoming hit*	1.367	0.572	5.721	3.924	1.280	12.03
Number of defenders involved in the tackle*	− 0.553	0.853	5.432	0.575	0.361	0.916

^a^aOR represents the difference in odds per point of the given variable

*95% confidence interval for odds ratio does not include 1

## Discussion

To expand on previous work examining the use of video analysis in concussion identification [4–8, 10], we explored the rates of six observable signs of concussion, match play characteristics, and how the combinations of these signs were able to predict a concussion diagnosis when the CIR was activated. Many variations of observable signs were present following a suspected concussion in NRL match play. Considered individually, positive anticipation of the oncoming hit, the number of defenders involved in the tackle, unresponsiveness or loss of consciousness at the time of injury, clutching or shaking the head at the time of injury, vacant stare at the time of injury, and seizure at the time of injury were potential predictor variables that led to a concussion diagnosis. Considered in combination, only positive anticipation of the oncoming hit, a fewer number of tacklers involved in the collision, and a vacant stare were significantly *independently* associated with a medical diagnosis of concussion. Using a cutoff threshold of three out of six observable on-field concussion signs to distinguish between those who were or were not medically diagnosed with concussion resulted in low sensitivity (0.30). Therefore, it was anticipated that further insight might be gained by examining which combinations of these types of signs may lead to improved identification accuracy over simply the total number of signs. However, no single combination of signs led to high levels of discriminative ability, likely due, at least in part, to the low number of observations for each category.

It is important to note that prior work reviewing an entire NRL season [4] identified that the six concussion signs occur commonly in NRL match play in the *absence* of the CIR being activated and in the *absence* of a diagnosed concussion. For example, the sign slow to get up was observed 2240 times over the course of the entire season, but of those 2240 times, only 223 instances looked like they may have been a concussion-related sign (10.0%) [4]. Of the 223 instances, 153 players were removed under the CIR and 60 were medically diagnosed as having a concussion [4]. In other words, all players medically diagnosed with a concussion that season (*N* = 60) were slow to get up, but this sign is very common in the absence of concussion. Similarly, prior work has reported that clutching the head is the most common observable sign among NRL players who used the CIR [6]; however, clutching the head also occurs commonly in the absence of the CIR being activated or a diagnosed concussion [4]. In addition, clutching the head was not significantly associated with a concussion diagnosis following CIR use in the current study. In contrast, the presence of a blank or vacant stare was a significant predictor of an eventual diagnosis of concussion in our study. Additionally, positive anticipation of an oncoming collision was independently associated with sustaining a concussion in those for whom the CIR was activated. It may be that the nature of the game and the nature of collisions and tackles differ between rugby league and other sports such as ice hockey that have demonstrated an association between *unanticipated* collisions and increased risk for concussion [16]. Anticipation of the impact is an interesting variable to consider across these sports. Unlike hockey, which is a 360° sport, rugby league tends to be a 180° sport, meaning a ball carrier does not often get “blindsided” by a tackler, and a tackler (almost all of the time) is engaged in making the contact and therefore is almost always anticipating contact. So despite there being few examples of a player not anticipating a hit in professional rugby league, very few of those events led to a subsequent diagnosed concussion. It is also possible that the significant anticipation of the hit is a spurious finding. It is also important to note that nearly two out of three tackles in the NRL are made by one or two defenders (i.e., 63% of tackles involved one or two defenders; 17 and 46%, respectively) [11]. Therefore, offensive players are exposed to a greater number of tackles, per season, by one or two players, so this greater exposure could underlie the independent association. Similarly, we speculate that a greater percentage of tackles over the course of the season also involve the offensive player anticipating the hit (versus not), and so the greater overall number of tackles in this scenario might underlie the association. In contrast, the technique used in a one-on-one tackle or a two-man tackle differs from that of a tackle involving three or more defenders, potentially resulting in an altered injury risk.

The sole reliance on video signs to identify a potential concussion can be problematic. In the National Hockey League (NHL) for example, visual signs have been found to differ in their ability to predict a diagnosis of concussion, and approximately 53% of the concussions were not associated with visual signs [3]. Therefore, examining other potential variables (i.e., mechanism of injury) in combination with various video signs may improve the sensitivity and specificity of identifying or predicting concussion [1]. Specifically, Bruce and colleagues [1] reported that suspected LOC and balance problems each account for unique variance in subsequent concussion diagnoses. Initial contact with the shoulder and secondary contact with the ice increased the risk of concussion diagnosis among athletes who exhibit a visual sign. The current study found that vacant stare, positive hit anticipation, and fewer number of tacklers involved in the tackle predicted concussion diagnoses in professional rugby league players.

There were several limitations of the current study. Not all signs were identified in every incident due to variability in the quality of the available video footage (i.e., in some instances, the view of the incident was obscured, or there was no closeup footage). These missing data were excluded from the analyses, which might have slightly improved the support for the utility of some of the visible signs. This issue was most commonly encountered when attempting to identify the blank or vacant stare sign. For these reasons, it is important to ensure that high-quality footage with the capability of multi-angle, slow motion replay, and narrow zoom options are available when reviewing video footage on the sideline [13]. Only one reviewer completed the coding of the events surrounding the use of the CIR for every game in the season; the inter-rater reliability of that type of coding is unknown. Additionally, the video reviewer was only partially blinded to the use of the CIR but completely blinded to the sideline assessment results and the medical diagnosis of concussion when reviewing the matches. Another study limitation was that there were no concussions that were subsequently diagnosed after the match day by the club medical personnel and reported to the researchers, which is inconsistent with other video review studies that have reported a numbers of cases of post-game diagnosis of concussion [3]. Our use of an operational definition of exclusion to categorize the observed signs into “plausible concussion signs” versus signs that were more likely attributable to other factors was also a limitation in terms of its subjectivity and reproducibility for future work. Further, in 16 instances, players were removed under the CIR, not diagnosed with a concussion, but did not return to play. Although data were not available regarding the reason for this, potential reasons may have been that they sustained an injury other than concussion that required their removal from gameplay or that the injury occurred near the end of the match. Importantly, the six signs that were reviewed in this study were signs that the researchers chose to observe, not necessarily the specific signs that the team medical personal who are making the in-game decisions on the sideline are also using to make their decisions to use the CIR. Finally, the current study was a post-game review of a men’s professional rugby league, and as such the results may not necessarily be generalizable to other levels of match play.

## Conclusion

Video replay analysis is useful for sports medicine professionals, but it also can be difficult to interpret and presents challenges in identifying those who have sustained a concussion. Advancements in the in-game detection of concussion may be improved by establishing clear definitions, providing education pertaining to observable signs of concussion, having an independent concussion expert on the sideline or in the stands who can identify players to be removed from play, and improving access to video replays, particularly systems that allow for multiple angle reviews, in addition to improving the communication between video observers and sideline medical personal.
